# The giant left atrium: when the heart outgrows the chest

**DOI:** 10.1093/ehjcr/ytag135

**Published:** 2026-02-28

**Authors:** Angelina Borizanova, Elena Kinova, Semra Beyti, Assen Goudev

**Affiliations:** Department of Emergency Medicine, Medical University Sofia, Byalo more str. 6, 1527 Sofia, Bulgaria; Clinic of Cardiology, UMHAT ‘Tsaritsa Yoanna-ISUL’ Byalo more str. 6, 1527 Sofia, Bulgaria; Department of Emergency Medicine, Medical University Sofia, Byalo more str. 6, 1527 Sofia, Bulgaria; Clinic of Cardiology, UMHAT ‘Tsaritsa Yoanna-ISUL’ Byalo more str. 6, 1527 Sofia, Bulgaria; Radiology, UMHAT ‘Tsaritsa Yoanna-ISUL’, Byalo more str. 6, 1527 Sofia, Bulgaria; Department of Emergency Medicine, Medical University Sofia, Byalo more str. 6, 1527 Sofia, Bulgaria; Clinic of Cardiology, UMHAT ‘Tsaritsa Yoanna-ISUL’ Byalo more str. 6, 1527 Sofia, Bulgaria

**Keywords:** Giant left atrium, Mass effect, Transthoracic echocardiography, Multimodality imaging

## Case description

A 76-year-old woman was evaluated preoperatively for excision of a right thigh skin tumour. Her history included severe mitral regurgitation in the setting of previously documented mitral valve prolapse (previously recommended surgery declined), permanent AF, and HF (NYHA II), with no history of rheumatic fever or endocarditis. Examination showed jugular venous distension, an irregular rhythm with a systolic murmur, reduced breath sounds at the left lung base, and mild oedema. NT-proBNP was elevated at 2898 pg/ml.

**Figure 1:**
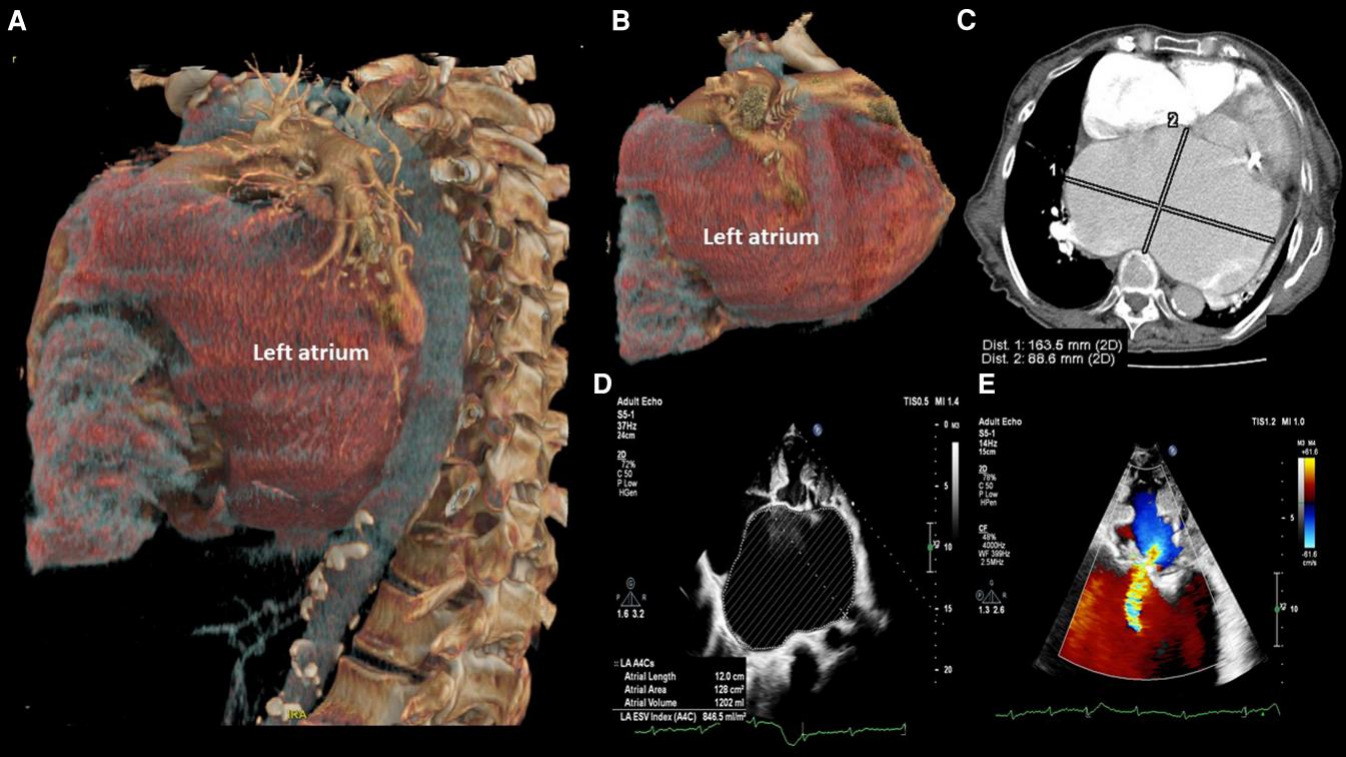
Multimodal imaging of a giant left atrium. *(A)* Computed tomography 3D reconstruction (left lateral view) highlighting the enlarged left atrium in contact with the spine, causing displacement of the oesophagus and descending aorta. Pulmonary vessels, left atrial appendage, left ventricle, and thoracic aorta are also visible. *(B)* Computed Tomography 3D reconstruction (left lateral view) highlighting the enlarged left atrium, shown without the spine, demonstrating deformation of the giant left atrium due to compression by the vertebral column. *(C)* Axial computed tomography image at the level of the mitral valve showing mitral annular calcification, predominantly involving the posterior mitral leaflet with left atrial dimensions of 163.5/88.6 mm. *(D)* Apical four-chamber view demonstrating a markedly increased left atrial volume index of 846.5 ml/m^2^. *(E)* Apical four-chamber transthoracic echocardiographic view showing thickened mitral valve leaflets consistent with degenerative changes, along with a central mitral regurgitation jet. Concentric left ventricular hypertrophy is also noted.

Transthoracic echocardiography demonstrated a GLA (anteroposterior diameter 105 mm; volume 1202 ml; indexed 846.5 ml/m^2^), thickened and restricted mitral leaflets with severe mitral annular calcification, and combined severe mitral stenosis (valve area 0.94 cm^2^, mean diastolic gradient 8 mmHg at heart rate of 97 b.p.m.) and severe mitral regurgitation (effective regurgitant orifice area 0.64 cm^2^, regurgitant volume 96 ml) (*Figure 1*). The left ventricle was dilated but with preserved ejection fraction. The right atrium was markedly enlarged, with severe functional tricuspid regurgitation due to a combined mechanism of advanced atrial dilation and leaflet thickening and associated pulmonary hypertension.

Contrast-enhanced CT confirmed massive left atrial enlargement compressing the oesophagus, descending aorta, and left lower lung lobe (*Figure 1*). Because of high surgical risk, the skin tumour was removed under local anaesthesia. One year later, after sustaining pelvic and hip fractures from a fall, the patient deteriorated and died under conservative management.

A giant left atrium is uncommon (∼0.3%) and traditionally linked to rheumatic mitral stenosis, where chronic pressure overload leads to progressive atrial dilatation.^1^ Non-rheumatic GLA, however, is increasingly recognized in degenerative, congenital, or mixed valvular disease and is typically driven by combined pressure–volume overload, often dominated by severe regurgitation.^2,3^ In our patient, the coexistence of mitral valve prolapse/dysplasia, extensive mitral annular calcification, and tricuspid valve abnormalities indicates a non-rheumatic aetiology suggesting a combined congenital and degenerative substrate, distinct from the classical rheumatic phenotype. Beyond chamber dilatation, GLA can exert mass effect on surrounding structures, producing dysphagia, hoarseness due to recurrent laryngeal nerve compression (Ortner's syndrome), and restrictive pulmonary symptoms.^4^ In this case, CT demonstrated significant displacement of the oesophagus and descending aorta and hypoventilation of the left lower lobe, despite minimal clinical symptoms.

These findings underscore the importance of timely intervention in significant mitral valve disease to prevent irreversible remodelling and compression-related complications. Multimodality imaging remains central for elucidating valvular pathology, evaluating the degree of atrial enlargement, and identifying its functional consequences.

## Supplementary Material

ytag135_Supplementary_Data

## Data Availability

All relevant clinical data supporting the findings of this case report are included within the main manuscript. No additional datasets or software code was generated or analysed. Due to the nature of this being a single-patient case report and in compliance with patient confidentiality, raw data are not publicly available. Further details can be made available from the corresponding author upon reasonable request and subject to institutional and ethical approval.

## Supplementary material

**Consent:** Written informed consent was obtained from the patient's next of kin for publication of this case and associated images.
